# Surgery

**Published:** 1878-09

**Authors:** 


### SURGERY.

SURGICAL VENOUS THROMBOSIS. M. Azam. (*Le Progrès Médical*, July 13, 1878, p. 543.)

The author read a paper to the *Société de Chirurgie* of which the following are the conclusions :

1st. Venous thrombosis of surgical origin is possible after fractures, contusions, phlebitis, varices, chronic inflammations in the neighborhood of large veins, and by the pressure upon the veins of rapidly developed tumors.

2nd. Venous thrombosis suspected by œdema of the parts situated below, is confirmed by direct exploration by the fingers along the course of the efferent veins.

3rd. The escape of migratory clots may be provoked by the movements of the patient, by extended exploration of the thrombotic veins, by massage of the limbs affected, or by the sudden suppression of the compression of a thrombotic vein.

4th. The accidents which are provoked by migratory clots are of various orders ; according to their size they are, malaise, pleurisies with limited effusion, partial pneumonias, spitting of blood, syncopies, asphyxia and sudden death.

5th. The surgeon may prevent the formation of venous clots by avoiding as much as possible compression of all the large veins, and by having the greatest regard for their internal membranes, in hastening the cure of neighboring, chronic or deep inflammations.

6th. If the thrombosis is confirmed, he should avoid the separation of the clots by opposing as far as possible local and general movements, and in emptying by successive punctures the collections of liquids in the vicinity of thrombotic veins.

M. Verneuil asked M. Azam to add sudden pyemia to his list of disasters from nervous thrombosis. He has seen some striking examples.

M. Tillaux remarked that certain emboli, the result of venous thrombosis, are arrested in the right heart, causing death by syncope. He has seen in a case of thrombosis of the femoral vein, sudden death caused by a clot lodged in the columns of the right ventricle.

Complete Resection of the Wrist: Conservation of the Movements of the Fingers and Hand. M. Reverdin. (*Le Progrès Médical*, July 13, 1878, p. 543.)

The author performed the operation upon a man of 41 years, in whom the member was compromised by a suppuratlve arthritis consecutive to a contused wound of the thumb. The operation was done after Lister's method, April 12th, 1877. M. Reverdin attacked the articulation by a simple dorsal incision in the neighborhood of the external border of the wrist; the necrosed bones of the wrist, the inferior extremities of the radius and ulna were extracted without much difficulty. The specimens were shown to the society. But one accident occurred—secondary hæmorrhage of the radial, after eight days, and M. Reverdin was obliged to tie both ends of the artery. Except this complication, the course was very simple.

To-day the patient has recovered the freedom of motion in the wrist; there is no loss of force in the muscles of the forearm. The three last fingers only present an incomplete mobility. However, the patient is able to use his hand for writing, and for some small carpentry work.

M. Reverdin, however, is not perfectly satisfied with the operative procedure. In order not to cut the extensor tendons, he had to draw them away forcibly. Perhaps it would be more simple, he adds, to cut one or more extensor tendons, because they remain too long after the operation, the regeneration of the bones always being incomplete.

CHOLECYSTOTOMY.—Sims.—(*Br. Med. Jour.*, June 8, 1878.) A long account of a very interesting case which lead to the above operation, the first of its kind, is given by Dr. Marion Sims. It occurred in the person of an American lady residing in Paris. Without giving the details we will only say that it was a case of obstruction of the duct with dropsy of the gall-bladder from gall stone. Considerable fluid had been removed by aspiration and there was no doubt as to the diagnosis of the cystic trouble. She had been seen by the most celebrated French physicians, who were able to offer no relief. The symptoms were most acute and death was imminent when Dr. Sims proposed and executed the following plan of operation: An incision, three inches long, parallel with the linea alba, and three inches to the right of the umbilicus, was made over the mcst prominent part of the tumor and down to the peritoneum. The whole operation was made under the antiseptic spray, and with all the usual precautions. After hæmorrhage had ceased and the peritoneum been opened, a trocar was thrust into the tumor caused by the distended gall-bladder and nearly 1,000 C. C. of dark brown fluid withdrawn. When the cyst was emptied it was hooked up with a tenaculum to the edge of the incision and drawn out with the fingers. It was then incised with scissors for about two inches, and thoroughly cleansed out with sponge porbangs; along with more viscid fluid, several gall stones were thus removed. Having thus swabbed out the gall-bladder, its open border was secured to the upper angle of the abdominal incision so as to secure a fistulous outlet. A projecting portion was amputated. After waiting for all hæmorrhage to cease, the abdominal wound was closed.

The patient's condition remained satisfactory for some days, but on the ninth day she sank, and died vomiting blood, and with hæmorrhage from mouth and nasal cavity.

Post-mortem inspection showed the abdominal wound everywhere healed, and no evidences of peritonitis. The gall-bladder was greatly thickened, and contained sixteen sacculated gall-stones. Death had been caused by hæmorrhage from the mouth and stomach, which had resisted all treatment and was probably due to changes in the blood.

So much for the conception and execution of a new operation by an American surgeon, to which he has given the most appropriate name as above. With aspiration as a means of diagnosis and antisepticism as a means of safety, there seems no reason why it should not take its place with ovariotomy and other similar operations as a *dernier resort*, and at the same time as a most positive relief in cases where it is appropriate.

Spontaneous Disintegration of Vesical Calculi.—At a meeting of the London Pathological Society (*British Medical Journal, June* 8, 1878), Dr. Ord exhibited several specimens illustrating the spontaneous disintegration of calculi within the bladder. All were fragments of fusiform calculi, which had been disrupted and then coated with urate of ammonia. They were evidently originally deposited from an acid urine; the urine had become more alkaline, and the outer layers were of less acid material. Owing to this change of environment, the nuclei had swollen and acted as a bursting charge. This accounted for nuclei being so rarely found in such cases.

Cystine Calculi.—Southam.—(*Br. Med. Jour.*, July 13, 1878.)

Out of a total number of fifty-five cases of vesical calculi, S. reports four cases where the stone was composed almost entirely of cystine—a very large proportion. Three of these cases came from the same locality, while two of them occurred in females. In one of them the condition of cystinuria persisted for several years. Two of the calculi were removed by lithotrity, one by lithotomy, and one by dilatation of the urethra (female).

